# Large phonon thermal Hall conductivity in the antiferromagnetic insulator Cu_3_TeO_6_

**DOI:** 10.1073/pnas.2208016119

**Published:** 2022-08-15

**Authors:** Lu Chen, Marie-Eve Boulanger, Zhi-Cheng Wang, Fazel Tafti, Louis Taillefer

**Affiliations:** ^a^Département de Physique, Institut Quantique and Regroupement Québécois sur les Matériaux de Pointe, Université de Sherbrooke, Sherbrooke, QC J1K 2R1, Canada;; ^b^Department of Physics, Boston College, Chestnut Hill, MA 02467;; ^c^Canadian Institute for Advanced Research, Toronto, ON M5G 1M1, Canada

**Keywords:** thermal Hall effect, antiferromagnetism, phonons, impurities, thermal conductivity

## Abstract

Phonons are known to generate a thermal Hall effect in certain insulators, including oxides with rare-earth impurities, quantum paraelectrics, multiferroic materials, and cuprate Mott insulators. In each case, a special feature of the material is presumed relevant for the underlying mechanism that confers chirality to phonons in a magnetic field. A fundamental question is whether a phonon Hall effect is an unusual occurrence—linked to special characteristics such as skew scattering off rare-earth impurities, structural domains, ferroelectricity, or ferromagnetism—or a much more common property of insulators than hitherto believed. To help answer this question, we have turned to a material with none of the previously encountered special features: the cubic antiferromagnet Cu_3_TeO_6_. We find that its thermal Hall conductivity $\kappa_{\text{xy}}$ is among the largest of any insulator so far. We show that this record-high $\kappa_{\text{xy}}$ signal is due to phonons, and it does not require the presence of magnetic order, as it persists above the ordering temperature. We conclude that the phonon Hall effect is likely to be a fairly common property of solids.

## Significance

Phonons are believed not to be able to generate a thermal Hall signal due to their lack of charge or spin. However, since the first discovery of a phonon thermal Hall effect in the paramagnetic insulator Tb_3_Ga_5_O_12_, much larger signals have been observed in several other families of insulators, which raises a fundamental question: How can phonons become chiral in a magnetic field? Most of the insulators that exhibit a phonon Hall effect have some special feature, believed to be a key to the underlying mechanism. Here, our discovery of a large phonon thermal Hall conductivity in a simple material with none of the special features of the previous cases opens up the subject into a much broader question.

The thermal Hall effect is the thermal analog of the electrical Hall effect. Instead of a transverse voltage induced by a perpendicular magnetic field in the presence of an electric current, a transverse temperature difference is induced in the presence of a heat current. The thermal Hall effect is a consequence of what we call “chirality”—which we define as a handedness that heat carriers acquire in a magnetic field. Electrons acquire chirality through the Lorentz force acting on charge carriers. However, understanding how chirality arises for electrically neutral particles—like phonons, magnons, or more exotic excitations—relies on new and mostly unknown mechanisms.

The phonon thermal Hall effect was first observed in the insulator Tb_3_Ga_5_O_12_ (1, 2), whose small thermal Hall conductivity $\kappa_{\text{xy}}$ was attributed to a special skew scattering of phonons by superstoichiometric Tb impurities (3). Later on, a much larger $\kappa_{\text{xy}}$ was measured in the multiferroic material Fe_2_Mo_3_O_8_, a ferrimagnetic insulator, where it was attributed to phonons in the presence of strong spin-lattice coupling (4). More recently, an even larger $\kappa_{\text{xy}}$ was reported in two other families of insulators: the cuprate Mott insulators (5–8), such as La_2_CuO_4_ and Sr_2_CuO_2_Cl_2_, and the quantum paraelectric SrTiO_3_ (9). There is little doubt that phonons are the bearers of chirality in both families, but the underlying mechanisms for the thermal Hall effect remain unknown.

The origin of phonon chirality is an open question. There are two classes of scenarios: scenarios based on the coupling of phonons to their pristine environment and scenarios based on the scattering of phonons by impurities or defects. For SrTiO_3_, the first type of scenario includes the flexoelectric coupling of phonons to their nearly ferroelectric environment, and the second type of scenario includes the scattering of phonons from structural domains (10). For cuprates, the first type includes the coupling of phonons to magnons (11) or spinons (12, 13), a special magnetoelectric order parameter (14), or an intrinsic fluctuating field arising from spin–lattice coupling (15). The second type includes the scattering of phonons by oxygen vacancies (16), by pointlike impurities in the presence of a Hall viscosity due to a coupling of phonons to their electronic environment (17), and by impurities and defects through the resonant skew-scattering process (18) or the “side-jump” effect (19).

In this article, we provide insights on the origin of phonon chirality by turning to a completely different and simpler material: Cu_3_TeO_6_. This is an insulator with a cubic structure, which retains its structure down to low temperature and therefore does not harbor structural domains. It also does not contain rare-earth elements and is neither a Mott insulator nor a multiferroic or nearly ferroelectric material. It develops three-dimensional long-range collinear antiferromagnetic order below the Néel temperature $T_{\text{N}}=63$ K (20, 21).

We report a thermal Hall conductivity among the largest ever observed in an insulator yet, with $\kappa_{\text{xy}}$ ≃1 W/K ⋅m at *T* = 20 K and *H* = 15 T in Cu_3_TeO_6_. This is 50 times larger than in the cuprate Sr_2_CuO_2_Cl_2_, for example. However, the phonon conductivity $\kappa_{\text{xx}}$ is also 50 times larger in Cu_3_TeO_6_, due to a better sample quality, resulting in less phonon scattering by defects and impurities. Then, the degree of chirality defined by the ratio $|\kappa_{\text{xy}}$/$\kappa_{\text{xx}}|$ is similar (in magnitude and temperature dependence) in these two very different materials with different structures, defects, and impurities. This shows that phonon chirality is a much more general phenomenon than hitherto perceived. Because $|\kappa_{\text{xy}}$/$\kappa_{\text{xx}}|$ goes smoothly through $T_{\text{N}}$, we infer that antiferromagnetic order per se is not required, but we speculate that a coupling of phonons to the spin degrees of freedom may nevertheless play a role.

## Results

**Fig. 1.**
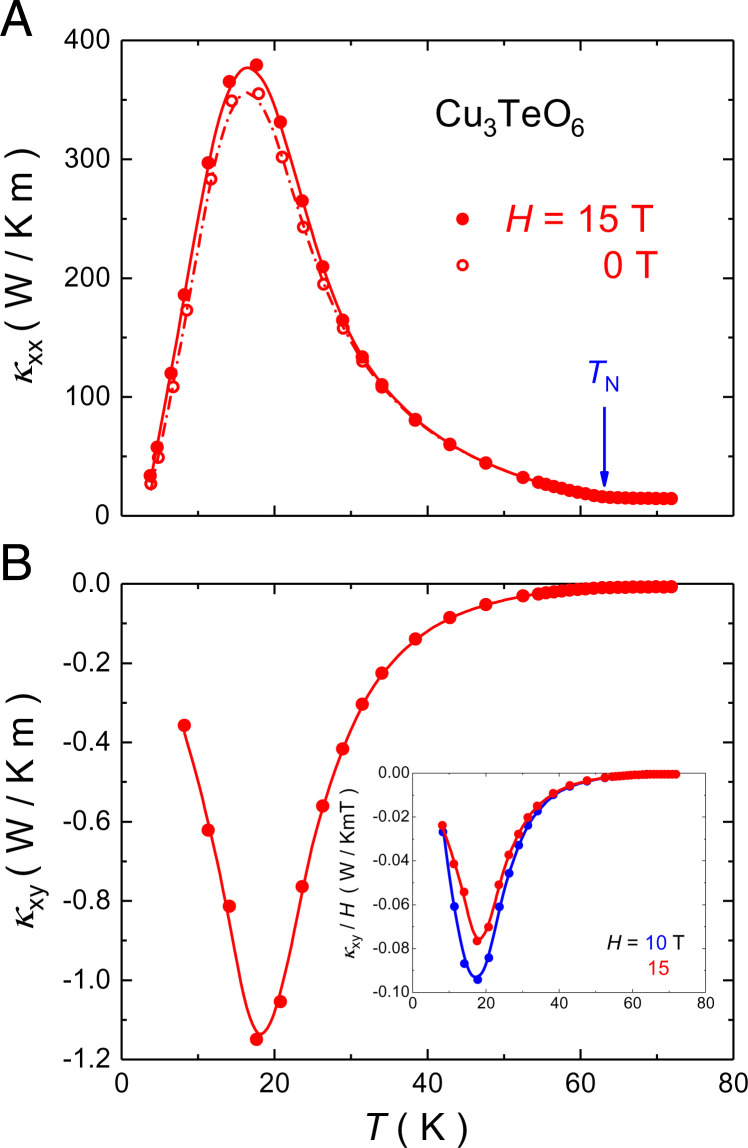
(*A*) Thermal conductivity $\kappa_{\text{xx}}$ of Cu_3_TeO_6_ as a function of temperature, in zero field (open circles) and in a magnetic field H=15 T (filled circles). The arrow marks the onset of antiferromagnetic order, at $T_{\text{N}}≃63$ K. (*B*) Corresponding thermal Hall conductivity $\kappa_{\text{xy}}$ at *H* = 15 T. *B*, *Inset* shows the *T* dependence of thermal Hall conductivity $\kappa_{\text{xy}}$ plotted as $\kappa_{\text{xy}}$/*H* at 10 T and 15 T. $\kappa_{\text{xy}}$ scales linearly with *H* above 40 K. Below 40 K, there is a slight sublinearity. Lines are a guide to the eye. Both $\kappa_{\text{xx}}(T)$ and $\kappa_{\text{xy}}(T)$ peak at T≃17 K, following a large increase relative to their values at $T_{\text{N}}$, by a factor of ∼25 and ∼150, respectively. The peak value, $|\kappa_{\text{xy}}|≃1.0$ W/K ⋅m, is among the largest thermal Hall conductivity reported to date in an insulator.

In Fig. 1*A*, the thermal conductivity $\kappa_{\text{xx}}$, measured at 0 T and 15 T, is plotted as a function of temperature. The field dependence of $\kappa_{\text{xx}}$ is weak, at most 6% (at T≃20 K) and negligible for *T* > 30 K (Fig. 1*A*). Our $\kappa_{\text{xx}}$ data are consistent with prior zero-field data (22), where the authors have argued that although magnons below $T_{\text{N}}$ can, in principle, carry heat, phonons dominate the thermal conductivity of Cu_3_TeO_6_, which is certainly the case above $T_{\text{N}}$. $\kappa_{\text{xx}}(T)$ shows a peak typical of phonons in insulators, located here at T≃20 K (Fig. 1*A*).

**Fig. 2.**
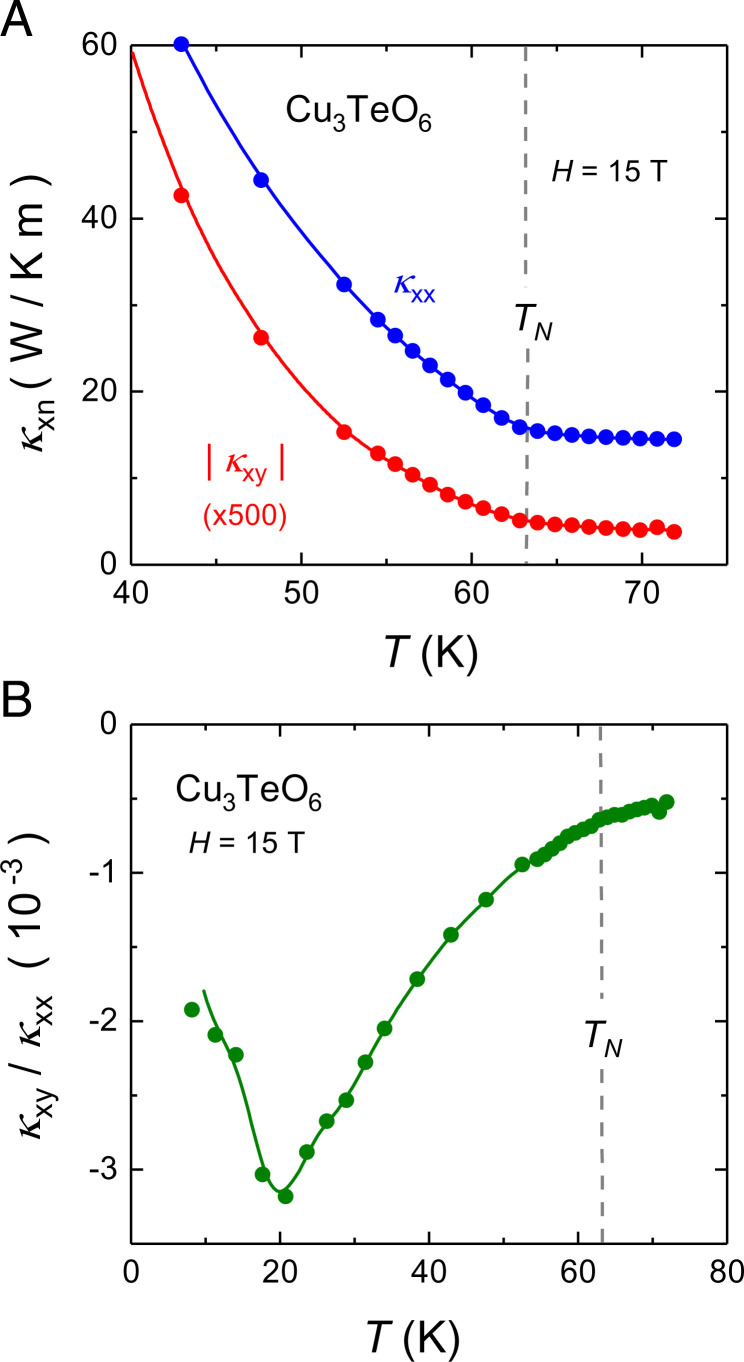
(*A*) Comparison of $\kappa_{\text{xx}}(T)$ (blue) and $\kappa_{\text{xy}}(T)$ (red; data multiplied by a factor of 500) near the antiferromagnetic transition at $T_{\text{N}}$ (dashed line). Both curves are seen to rise upon cooling below $T_{\text{N}}$. (*B*) Ratio of $\kappa_{\text{xy}}(T)$ over $\kappa_{\text{xx}}(T)$, vs. *T*, at *H* = 15 T. The magnitude of this ratio increases upon cooling from *T* = 70 K to *T* = 20 K. The fact that it goes smoothly through $T_{\text{N}}$ (dashed line) shows that the onset of long-range magnetic order does not directly affect the thermal Hall effect. Although $\kappa_{\text{xy}}$ in Cu_3_TeO_6_ is exceptionally large, the maximal value of the ratio, $|\kappa_{\text{xy}}/\kappa_{\text{xx}}|≃3\times 10^{-3}$, is typical of various insulators (Table 1). Lines are a guide to the eye.

In Fig. 2*A*, we zoom on the data near $T_{\text{N}}$. Above $T_{\text{N}},\;\kappa_{\text{xx}}$ is flat, evidence that phonons are scattered by spin excitations associated with the proximate onset of antiferromagnetic order (22). Upon cooling below $T_{\text{N}},\;\kappa_{\text{xx}}$ suddenly rises, presumably because that scattering is weakened when order sets in.

In Fig. 1*B*, the thermal Hall conductivity $\kappa_{\text{xy}}$, measured on the same sample in the same conditions, is displayed as a function of temperature. There is a large negative Hall effect. We see that $\kappa_{\text{xy}}(T)$ mirrors the evolution of $\kappa_{\text{xx}}(T)$, with a peak at the same temperature. As pointed out in ref. 9, this suggests that $\kappa_{\text{xy}}$ is carried predominantly by phonons, as is $\kappa_{\text{xx}}$. At its peak, $|\kappa_{\text{xy}}|≃1.0$ W/K ⋅m, among the largest values of $\left|\kappa_{\text{xy}}\right|$ reported so far in insulators (Table 1).

**Table 1.** Thermal Hall conductivity in various insulators

	$\kappa_{\text{xy}}$,	$\kappa_{\text{xx}}$,	$\kappa_{\text{xy}}/\kappa_{\text{xx}}$,	*T*, *H*;
Material	mW/K ⋅m	W/K ⋅m	10^–3^	K, T
Cu_3_TeO_6_	–1,000	330	–3.0	20, 15
Fe_2_Mo_3_O_8_ (4)	12	2.5	4.8	65, 14
Tb_2_Ti_2_O_7_ (23)	1.2	0.27	4.4	15, 12
Y_2_Ti_2_O_7_ (23, 24)	0	18	0	15, 8
La_2_CuO_4_ (5)	–38	12	–3.2	20, 15
Sr_2_CuO_2_Cl_2_ (6)	–21	7	–3.0	20, 15
Nd_2_CuO_4_ (6)	–200	56	–3.6	20, 15
SrTiO_3_ (9)	–80	36	–2.2	20, 12
KTaO_3_ (9)	2	32	0.06	30, 12
RuCl_3_ (25)	2	2	1.0	20, 15

The values of $\kappa_{\text{xy}}$ and $\kappa_{\text{xx}}$ are taken at the specified temperature *T* and field *H*. Their ratio gives the degree of chirality.

In Fig. 2*A*, we see that $\kappa_{\text{xy}}(T)$ evolves in tandem with $\kappa_{\text{xx}}(T)$ across $T_{\text{N}}$: It is almost flat above $T_{\text{N}}$ and rises below $T_{\text{N}}$. This parallel evolution is further evidence that $\kappa_{\text{xy}}$ is carried by phonons. It is instructive to plot the ratio $\kappa_{\text{xy}}$/$\kappa_{\text{xx}}$ vs. *T*, as done in Fig. 2*B*, a quantity that may be viewed as the degree of chirality—the extent to which phonons respond asymmetrically to a magnetic field. We see that the ratio goes smoothly through $T_{\text{N}}$, unaltered by the onset of antiferromagnetic order. This shows that long-range order per se does not play a key role in conferring chirality to phonons. Note that despite the high amplitude of $\left|\kappa_{\text{xy}}\right|$ in Cu_3_TeO_6_, the ratio $\kappa_{\text{xy}}$/$\kappa_{\text{xx}}$ is similar to that found in several other insulators (Table 1), as we discuss below.

## Discussion

In the antiferromagnetic insulator Cu_3_TeO_6_, two types of neutral excitations can be expected to generate a thermal Hall effect: magnons and phonons. We can rule out magnons, based on our empirical evidence and for theoretical reasons. Empirically, the fact that $\kappa_{\text{xy}}(T)$ mirrors the temperature evolution of the phonon-dominated $\kappa_{\text{xx}}(T)$ so well (Figs. 1 and 2*A*) argues against a large contribution to $\kappa_{\text{xy}}$ from magnons. Moreover, the fact that the degree of chirality, measured by the ratio $\kappa_{\text{xy}}$/$\kappa_{\text{xx}}$, goes through $T_{\text{N}}$ without anomaly (Fig. 2*B*) shows that long-range order, and therefore well-defined magnons, play little role in $\kappa_{\text{xy}}$. Theoretically, it has been shown that magnons can produce a thermal Hall effect in antiferromagnetic insulators, but only under certain conditions (26). In a collinear antiferromagnet, a condition is the presence of spin canting due to the Dzyaloshinskii–Moriya (DM) interaction. Now in Cu_3_TeO_6_, theoretical calculations and inelastic neutron scattering experiments show that the collinear antiferromagnetic ground state can be well understood by considering the antiferromagnetic exchange interactions and a global single-ion anisotropy term without introducing any DM interaction (27). Neutron powder diffraction results indicate that the possible noncollinear canting of spins is no more than 6^∘^ (20). Under such conditions, the $\kappa_{\text{xy}}$ signal expected from magnons is estimated to be much smaller than the signal reported in La_2_CuO_4_ (12), which is, in turn, much smaller than what we observe in Cu_3_TeO_6_. Theory also predicts a sizable thermal Hall effect of magnons in collinear antiferromagnetic insulators with a honeycomb lattice in which there is a spin–flop phase transition (28). However, neither of these features is present in the cubic Cu_3_TeO_6_ (20). So, the large thermal Hall conductivity in Cu_3_TeO_6_ is not generated by magnons.

This is in contrast to the case of VI_3_, an insulator for which magnons were recently shown to generate a thermal Hall effect (29), because of the large intrinsic DM interaction and associated magnon Berry curvature in that material. The difference between VI_3_ and Cu_3_TeO_6_ shows up clearly in the *H* dependence of $\kappa_{\text{xy}}$: $\kappa_{\text{xy}}$ is independent of *H* in the former and present at H≃0, whereas it is roughly linear in *H* in the latter (Fig. 1 *B*, *Inset*), as in other cases of phonon-mediated thermal Hall effect, including cuprates (6).

We also rule out that $\kappa_{\text{xy}}$ in Cu_3_TeO_6_ is generated by topological magnons, as is the case in the material VI_3_ (29). The topological magnons present in Cu_3_TeO_6_ are at high energy, namely, 17.75 meV (∼200 K) (27, 30). At T∼20 K, where $\kappa_{\text{xy}}$ in Cu_3_TeO_6_ is maximal, these topological magnons are not thermally excited, and the thermal transport is dominated by low-energy acoustic phonons that are not coupled to these topological magnons. These low-energy phonons are coupled to magnons, but nontopological ones at lower energy. So, we conclude that the Dirac magnons in Cu_3_TeO_6_ do not contribute to the measured thermal Hall effect in this material at low temperature.

The only type of heat carriers left that could generate a thermal Hall effect in Cu_3_TeO_6_ are phonons. Two empirical observations confirm that it is indeed the phonons that generate the huge $\kappa_{\text{xy}}$ in Cu_3_TeO_6_. First, $\kappa_{\text{xy}}(T)$ and $\kappa_{\text{xx}}(T)$ evolve in parallel across the antiferromagnetic transition, both increasing in tandem upon cooling below $T_{\text{N}}≃63$ K (Fig. 2*A*). Second, while the degree of chirality in Cu_3_TeO_6_ is not exceptionally high, what is exceptionally high among the insulators for which a $\kappa_{\text{xy}}$ signal has been reported is the phonon-dominated $\kappa_{\text{xx}}$ (Table 1). $\kappa_{\text{xy}}$ in Cu_3_TeO_6_ is very large, simply because $\kappa_{\text{xx}}$ is very large. This is a major finding of this work: The magnitude of $\kappa_{\text{xy}}$ from phonons scales with the magnitude of $\kappa_{\text{xx}}$. Note also that the field dependence of $\kappa_{\text{xy}}$ in Cu_3_TeO_6_, displayed in Fig. 1 *B*, *Inset*, is very similar to that of cuprates (6), materials for which the thermal Hall effect is known to arise from phonons (7). So, the record-high thermal Hall conductivity in Cu_3_TeO_6_ is a property of phonons.

The remaining question is: What makes phonons chiral in Cu_3_TeO_6_ ? Several authors have proposed that the phonon thermal Hall effect is based on the scattering of phonons by impurities (or defects) (16, 18, 19). This may well be part of the answer. In support of an impurity-based mechanism, we note that $\left|\kappa_{\text{xy}}(T)\right|$ is maximal at the temperature where $\kappa_{\text{xx}}$ (*T*) peaks (Fig. 1)—namely, at 20 K, the temperature where impurities (or defects) are expected to be the dominant scattering process. Moreover, the degree of chirality—the ratio $|\kappa_{\text{xy}}$/$\kappa_{\text{xx}}|$—is maximal at that temperature (Fig. 2*B*). Note that boundary scattering is the dominant scattering process at the lowest *T*, with the phonon mean free path (31) approaching the size of our sample at T→0. However, the mean free path is much shorter at 20 K, by a factor ∼20, due to other scattering processes. The obvious process is scattering by local defects, since the phonons that dominate the thermal conductivity have an energy ∼4 $k_{B}T$ and, thus, a wavelength of ∼4 Å at 20 K—the size of a local defect.

However, within such a class of scenarios, a number of puzzles remain. First, it is clear that not all impurities can generate a phonon thermal Hall effect. For example, there is a $\kappa_{\text{xy}}$ signal in Tb_2_Ti_2_O_7_, but not in the closely related material Y_2_Ti_2_O_7_ (32). Here, the mechanism is perhaps the presence of Tb ions as superstoichometric impurities that act as skew scatterers, as argued for Tb_3_Gd_5_O_12_ (3). The question then is: What type of impurity (or defect) can give rise to phonon chirality?

It may be that one should consider not just the impurity, but, rather, the effect of that impurity (or defect) on its local environment. For example, there is a phonon $\kappa_{\text{xy}}$ signal in the cuprate Nd-LSCO at a doping *p* = 0.20, but not in the same material at *p* = 0.24 (5, 7). There is no significant difference between the two dopings in terms of impurities or defects. But the two dopings correspond to different states of matter: the pseudogap phase in the former $\left(p<p^{⋆}\right)$ and the strange metal phase in the latter $\left(p>p^{⋆}\right)$. So here, it appears that it is the nature of the environment that matters, not the nature of the impurity or defect.

**Fig. 3.**
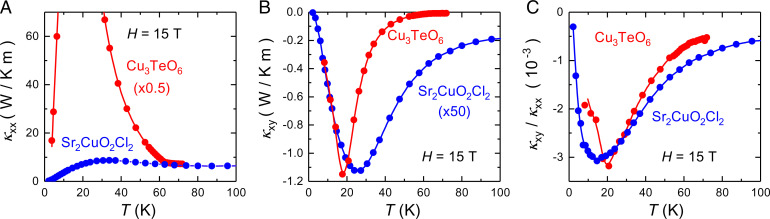
Comparison of two antiferromagnetic insulators, whose thermal transport was measured in a magnetic field *H* = 15 T: Cu_3_TeO_6_ (red; this work) and the cuprate Mott insulator Sr_2_CuO_2_Cl_2_ [blue (6)]. (*A*) $\kappa_{\text{xx}}$ vs. *T*; the data for Cu_3_TeO_6_ are multiplied by a factor of 0.5. (*B*) $\kappa_{\text{xy}}$ vs. *T*; the data for Sr_2_CuO_2_Cl_2_ are multiplied by a factor of 50. (*C*) $\kappa_{\text{xy}}$/$\kappa_{\text{xx}}$ vs. *T*; no multiplicative factor. All lines are a guide to the eye.

A second puzzle is the fact that the ratio $|\kappa_{\text{xy}}$/$\kappa_{\text{xx}}|$ has a similar magnitude (at the peak temperature) in a wide variety of materials with very different types and levels of impurities and defects—namely, $|\kappa_{\text{xy}}$/$\kappa_{\text{xx}}|=3-5\times 10^{-3}$ (at *H* = 15 T) (Table 1). In this respect, it is interesting to compare Cu_3_TeO_6_ with Sr_2_CuO_2_Cl_2_, as done in Fig. 3. In Fig. 3*C*, we see that the ratio $\kappa_{\text{xy}}$/$\kappa_{\text{xx}}$ has the same sign, magnitude, and temperature dependence in these two very different materials, even though the level of impurity or defect scattering in the two samples is clearly very different. Indeed, the peak value of $\kappa_{\text{xx}}$ is 50 times larger in Cu_3_TeO_6_.

A third puzzle arises from this comparison. If the phonon thermal Hall effect is caused by impurity scattering, how can the ratio $\kappa_{\text{xy}}$/$\kappa_{\text{xx}}$ remain the same in the two materials at all temperatures (Fig. 3*C*), given that in one case (Cu_3_TeO_6_), the dominant phonon-scattering process goes from impurities at *T* = 20 K to spin fluctuations (and other phonons) at *T* = 70 K, whereas in the other case (Sr_2_CuO_2_Cl_2_), it remains impurities at *T* = 70 K? This difference shows up in the *T* dependence of $\kappa_{\text{xx}}$ (Fig. 3*A*)—nearly constant in Sr_2_CuO_2_Cl_2_ and dropping by a factor 20 to 25 in Cu_3_TeO_6_.

All these suggest that the nature of the environment in which impurities are embedded and phonons propagate matters. We suggest that a magnetic environment—namely, the presence of spins (but not long-range order)—may be relevant. Several authors have shown theoretically that a phonon thermal Hall effect can arise from a coupling of phonons to spins (11, 33–37).

## Summary and Outlook

We have measured the thermal conductivity $\kappa_{\text{xx}}$ and the thermal Hall conductivity $\kappa_{\text{xy}}$ of the antiferromagnetic insulator Cu_3_TeO_6_. We report a value of $\left|\kappa_{\text{xy}}\right|$ that is among the largest ever observed in an insulator. We provide arguments for why $\kappa_{\text{xy}}$ must be due to phonons, and not magnons. Arguments in favor of phonons include: 1) $\kappa_{\text{xy}}$ (T) and $\kappa_{\text{xx}}$ (T) peak at the same temperature; 2) $\kappa_{\text{xy}}$ and $\kappa_{\text{xx}}$ evolve in the same way across the antiferromagnetic (AFM) ordering temperature $T_{\text{N}}$; and 3) $\kappa_{\text{xy}}$ scales in magnitude with the phonon-dominated $\kappa_{\text{xx}}$ across various materials, including Cu_3_TeO_6_. Arguments against magnons include: 1) a magnon Hall effect is ruled out for a collinear AFM order (26); 2) topological magnons are at very high energy (27) and thus irrelevant to the low-energy thermal transport; and 3) a sizable $\kappa_{\text{xy}}$ is observed above $T_{\text{N}}$, where there are no magnons.

On the basis of a comparison with the cuprate material Sr_2_CuO_2_Cl_2_, which exhibits the same ratio $\kappa_{\text{xy}}$/$\kappa_{\text{xx}}$, or degree of chirality, both in magnitude and in temperature dependence, even though its phonon conductivity is 50 times smaller, we conclude that the mechanism for phonon chirality could involve the scattering of phonons by impurities or defects. But this scattering process would depend on the nature of the environment in which the impurities or defects are embedded. Although the nature of this coupling remains unclear, we propose that a likely possibility is scattering from local spin texture created by an impurity or defect embedded in a magnetic environment, not necessarily with long-range order. Our findings suggest that a large phonon thermal Hall effect may be a common occurrence in magnetic insulators.

Our findings put two prior studies of the thermal Hall effect into perspective. First, they raise the question of whether the $\kappa_{\text{xy}}$ signal measured in the Kitaev material *α*-RuCl_3_ (38), hitherto attributed entirely to Majorana fermions (39), may in part be due to phonons (25). In particular, our finding that the magnitude of $\kappa_{\text{xy}}$ scales with the magnitude of $\kappa_{\text{xx}}$ nicely explains why $\kappa_{\text{xy}}$ in *α*-RuCl_3_ varies so much from sample to sample (25, 39–42)—a natural variation in a phonon scenario, where phonon conduction varies due to the sample quality. Secondly, our findings raise the possibility that the phonon thermal Hall conductivity in hole-doped cuprates that appears upon entering the pseudogap phase (5, 7) may be the signature of short-range antiferromagnetic correlations. Data on electron-doped cuprates are consistent with short-range antiferromagnetic correlations playing a role in inducing $\kappa_{\text{xy}}$ (8).

## Materials and Methods

Single crystals of Cu_3_TeO_6_ were grown from CuO powder and TeO_2_ flux. The starting materials were mixed in a molar ratio of 3:5 and heated to 870 ^∘^C at 5 ^∘^C/min, held for 24 h, cooled to 700 ^∘^C at 1.5 ^∘^C/h, and cooled to room temperature at 3 ^∘^C/min. Crystals of approximate dimensions 4×4×1 mm^3^ were harvested after washing the solvent with sodium hydroxide and deionized water. Our sample was cut and polished in the shape of a rectangular platelet, with the following dimensions (length between contacts × width × thickness): (2.2±0.1)×(1.61±0.04)×(0.07±0.01) mm. Cu_3_TeO_6_ has a centro-symmetric cubic crystal structure (20). It is not known to undergo any structural transition. The normal to each of the three faces of the sample is along each of the three equivalent high-symmetry (100) directions of the cubic lattice. Contacts were made by using silver wires and silver paint. The thermal conductivity $\kappa_{\text{xx}}$ and thermal Hall conductivity $\kappa_{\text{xy}}$ were measured as described in refs 5, 6, and 43, by applying a heat current along the *x* axis (longest sample dimension) and a magnetic field along the *z* axis (normal to the largest face) and measuring the longitudinal ($\Delta T_{\text{x}}$) and transverse ($\Delta T_{\text{y}}$) temperature differences with type-E thermocouples.

## Data Availability

All study data are included in the article.
